# Association between dietary acid load and the risk of hypertension among adults from South China: result from nutrition and health survey (2015–2017)

**DOI:** 10.1186/s12889-019-7985-5

**Published:** 2019-11-29

**Authors:** Shao-wei Chen, Gui-yuan Ji, Qi Jiang, Ping Wang, Rui Huang, Wen-jun Ma, Zi-hui Chen, Jie-wen Peng

**Affiliations:** Department of Health Risk Assessment Research Center, Guangdong Provincial Institute of Public Health, Guangdong Provincial Center for Disease Control and Prevention, No. 160 Qunxian Road, Panyu District, Guangzhou, 511430 China

**Keywords:** Hypertension, Dietary acid load, Nutrition and health survey, China

## Abstract

**Background:**

Higher dietary acid load (DAL) was considered to be associated with an elevated risk of hypertension, while related data from mainland China remains scarce and incomplete. We aim to evaluate the association between DAL and the risk of hypertension among adults from South China.

**Methods:**

We conducted a nutrition and health survey in Guangdong Province located in southern China from 2015 to 2017. A four-stage probability sampling method was utilized to select representative samples of citizens aged ≥18 years old. DAL was assessed by potential renal acid load (PRAL) and net endogenous acid production (NEAP). Participants were divided to 4 groups (Q1-Q4) according to the quartile points of PRAL or NEAP distributions. Generalized linear mixed effects models were applied to evaluate the association between DAL and the risk of hypertension.

**Results:**

A total of 3501 individuals were eligible for this study and 45.9% was male participants. Hypertension rate was 30.7%. A higher PRAL was associated with higher prevalence rate of hypertension among the male (P-trend = 0.03). OR for Q2 was 1.34 (95%CI, 0.94–1.91), Q3 was 1.53 (95%CI = 1.08, 2.16) and Q4 was 1.51 (95%CI, 1.08–2.16) among the male. However, as for total participants, the female, the participants with ≤55 years or participants with > 55 years, the associations were lack of significance. With respect to association between NEAP and hypertension, non-significant results were identified.

**Conclusions:**

The current study indicated male hypertension was associated with higher PRAL, while given to this study was cross-sectional design, further studies are warranted to verify the association.

## Background

Hypertension is a leading cause of premature death around the world on account of its high prevalence and significant impact on cardiovascular disease mortality [1–3]. Large-scale and the most recent surveys exhibit that hypertension is commonly prevalent in China and its prevalence rate is up to 30% in adults [4]. Findings from previous studies have estimated that above 750,000 cardiovascular disease deaths could be attributable to abnormally high blood pressure in China in 2010 [5]. In addition, situation about hypertension control is not so optimistic for hypertension treatment rate is < 50% [5]. It is paramount and indispensable to prevent the hypertension from its etiological factors. For the etiology of hypertension, besides genetic, social and environmental factors, diet pattern could also exert a role in the ongoing epidemics of hypertension [6–10].

There are both potentially adverse and preventive effects of dietary factors on hypertension. Some previous publications [6, 11, 12] showed an inverse risk of developing hypertension associated with high quality and/or health-related dietary patterns, such as Mediterranean diets. These dietary patterns are perceived to be associated with inflammation suppression, which are involved in biological pathways of preventive effect on hypertension [13]. Another possible mechanism comes to endogenous acid and base production [14]. Individuals with highly consuming plant food are perceived to get a more sound equilibrium of acid-base as consumption of these food generally could obtain more alkali-rich nutrients such as magnesium and potassium [14]. The alkali-rich nutrients help to produce potential bicarbonate precursors in vivo [15]. However, people with habitually animal products might be much easier to get a chronic and mild metabolic acidosis because phosphorus-rich and sulfur-rich components from these food could provide proton load and lower pH value in blood [15, 16].

Several studies revealed that acid-base equilibrium affects blood pressure toward kidneys and renin-angiotensin-aldosterone system [15, 16]. Dietary acid load (DAL) was proposed to evaluate acid-base equilibrium resulted from dietary intake [17–19]. Two methods were commonly applied for computing DAL, including potential renal acid load (PRAL) and net endogenous acid production (NEAP) [17–19]. Some observational studies exhibited a higher risk of hypertension in citizens with higher DAL [14, 20, 21], but it is still under discussion as some other studies reported a contradictory result [22]. Up to now, to our best understanding, there is still lack of data from mainland China.

Guangdong province locates in the south of China and has a population of 110 million in 2016. We have previously reported the prevalence trend of hypertension from 23.6 to 40.8% within a period from 2002 to 2010 [23]. Risk factors of hypertension such as lifestyle, industrialization and urbanization were also studied [23], but data on dietary impacts were lacking. Thus, we perform a cross-sectional health survey from 2015 to 2017 including a population of 3501 in Guangdong province to explore whether DAL is associated with the risk of hypertension.

## Methods

### Study design

This study employed data from the Guangdong Nutrition and Health Survey (NHS) 2015, which was performed by the Guangdong Provincial Center for Disease Control and Prevention from 2015 to 2017. The NHS was cross-sectional design and was a part of the China National Nutrition and Health Survey 2015, as previously described [24]. A four-stage probability sampling method was utilized to select representative samples of citizens who lived in Guangdong province for continuous 6 months within the last year. A total of 14 counties in Guangdong province were selected and the procedure was similar to the NHS conducted in 2002 and 2012 [23, 25]. Briefly, 125 counties in Guangdong province were stratified into urban or rural areas according to economic capabilities. Eight counties from urban layer and six counties from rural layer were randomly selected. After then, 3 communities (urban) or townships (rural) were randomly sampled from each selected county. Two residential committees (urban) or villages (rural) were further extracted from the target communities or townships. Finally, about 20 households were included and all the adults aged ≥18 years old in the households attended our investigations with signed informed consent. To obtain sufficient number of participants, about 270 households and 612 participants in each county were required. Additional households and participants were obtained from neighboring area, if it did not meet the requirement in the specific selected county.

### Dietary consumption assessment

A 24-h dietary recall in consecutive 3 days was applied to collect dietary consumption at the individual level. Food species and intake amount were recorded by trained interviewers. Cooking oil and condiments at the household were weighted on daily basis. Individual data about cooking oil and condiments was calculated by individual ratio of dietary energy among household members. Questionnaire applied for collecting food consumption in this study was developed from the China National Nutrition and Health Survey 2015, as described in previous study [24]. Daily dietary nutrients and energy were computed by food consumption and food composition data which was derived from the Chinese Food Composition Tables (2004 and 2009). DAL was assessed by PRAL and NEAP. PRAL evaluated endogenous acid load though synthesizing effect of dietary protein, phosphorus, potassium, calcium and magnesium, and NEAP though dietary protein and potassium. PRAL and NEAP were computed using the following formulas, respectively [17–19]:

$$ \mathrm{PRAL}\ \left(\mathrm{mEq}/\mathrm{d}\right)=0.4888\times \mathrm{protein}\ \mathrm{intake}\ \left(\mathrm{g}/\mathrm{d}\right)+0.0366\times \mathrm{phosphorus}\ \left(\mathrm{mg}/\mathrm{d}\right)-0.0205\times \mathrm{potassium}\ \left(\mathrm{mg}/\mathrm{d}\right)-0.0125\times \mathrm{calcium}\ \left(\mathrm{mg}/\mathrm{d}\right)-0.0263\times \mathrm{magnesium}\ \left(\mathrm{mg}/\mathrm{d}\right); $$

$$ \mathrm{NEAP}\ \left(\mathrm{mEq}/\mathrm{d}\right)=\left(54.5\times \mathrm{protein}\ \mathrm{intake}\ \left(\mathrm{g}/\mathrm{d}\right)\div \mathrm{potassium}\ \mathrm{intake}\ \left(\mathrm{mEq}/\mathrm{d}\right)\right)-10.2 $$

### Anthropometry

Anthropometry of participants were measured by well-trained researchers based on standard procedures. Weight (kg) and height (cm) of participants were assessed by electronic instruments after removing the heavy clothes and shoes. Body mass index (BMI) was computed for each individual (weight kg/height m^2^). Systolic blood pressure (SBP) and diastolic blood pressure (DBP) were measured by an electronic device (OMRON Corporation, HBP1300) for 3 times in each participant. We applied the mean of the 3 measured values in the final analysis. According to guidelines of the World Health Organization (WHO), hypertension was defined as a SBP ≥ 140 mmHg and/or a DBP ≥ 90 mmHg, or the use of medication for hypertension [26]. Baseline information and hypertension related factors such as smoking, alcohol intake, physical activity and etc. were also collected by face to face interview.

### Statistical analysis

Collation of the data from all participants was conducted by 2 investigators. Data on continuous variables was exhibited as mean and standard deviation (SD), and data on categorical variables was as number and proportion. If there was abnormal or incomplete data, we excluded them from analysis of association between DAL and hypertension. Participants were divided to 4 groups according to the quartile points of DAL (PRAL and NEAP) distributions. Correlation between blood pressure (SBP and DBP) and different nutrients intake (protein, calcium, potassium, phosphorus and magnesium) was evaluated by scatter plot. We employed analysis methods for complex samples as the data in this study was collected by multi-stage complex sampling. A generalized linear mixed effects model was employed to examine the relationship between DAL and hypertension. As participant selection was stratified into urban or rural areas, we put region layers (urban layer or rural layer) as fixed effect in the model. Considering there might be family cluster of hypertension and food consumption, household id was put as random effect in the model. Crude odds ratio (OR) and its corresponding 95% confidence interval (CI) for difference levels of DAL (PRAL or NEAP) were calculated. In addition, as there were potential confounding factors for hypertension, including age, sex, smoking, drinking, BMI, sedentary leisure time, physical activity time, sodium intake, education and marital status, we also put these variables in the model to compute adjusted OR. Adjusted model 1 was deemed as generalized linear mixed effects model adjusted for sex and age, and adjusted model 2 was generalized linear mixed effects model adjusted for sex, age, smoking, drinking, body mass index, sedentary leisure time, physical activity time, sodium intake, education and marital status. Subgroups analysis concerning different gender groups (male and female) and age groups (≤55 years and > 55 years) was performed. Statistical analyses in this study were carried out by SAS Enterprise Guide (SAS Institute Inc., Cary, NC, USA). Figures were produced in R version 3.5.1 (R Core Development Team). A *p* value below 0.05 was conducted to characterize statistically significant results.

## Results

### Characteristic of participants

A total of 3643 adults was included in our study and 3501 adults completed the survey and anthropometry measurements. The response rate was 96.1%. Among the 3501 individuals, their average age was 52.0 ± 15.0 years and 45.9% was male participants. Prevalence of hypertension was 30.7% (29.1% in male and 32.7% in female). The mean value of PRAL and NEAP were 22.1 ± 18.6 and 86.8 ± 53.9 mEq/d, respectively. Nutrient intake and other variables about characteristic of participants are summarized in Table 1. A total of 3237 participants and 3233 participants were included in generalized linear mixed effects model for PRAL and NEAP analysis, respectively, because 268 participants and 264 participants had abnormal high value of PRAL and NEAP or hypertension patients were not under control after taking antihypertensive drugs.

**Table 1:** Characteristic of eligible participants

	Overall (*n* = 3501)	Male (*n* = 1893)	Female (*n* = 1608)
Age (years)	52.0 ± 15.0	52.9 ± 15.2	51.2 ± 14.7
Ethnicity (N, %)			
*Han*	3473(99.2)	1874(99.0)	1599(99.4)
*Other*	28(0.8)	19(1.0)	28(0.6)
BMI (Kg/m2)	23.4 ± 3.5	23.3 ± 3.4	23.4 ± 3.6
Nutrient intake			
*Protein (g/d)*	65.9 ± 36.5	71.5 ± 40.4	61.1 ± 32.0
*Calcium (mg/d)*	1501.6 ± 603.9	1564.6 ± 579.3	1448.1 ± 619.1
*Potassium (mg/d)*	381.1 ± 211.1	389.4 ± 184.7	374.1 ± 231.0
*Phosphorus (mg/d)*	866.6 ± 298.7	926.2 ± 298.9	815.9 ± 289.1
*Magnesium (mg/d)*	238.4 ± 82.3	250.7 ± 83.3	228.0 ± 80.0
*Sodium (mg/d)*	4893.5 ± 3366	5228.2 ± 3400.4	4609.2 ± 3310.9
Total energy intake (Kcal/d)	1757.4 ± 566.5	1908.6 ± 604.8	1629.0 ± 497.1
PRAL (mEq/d)	22.1 ± 18.6	25.3 ± 20.2	19.4 ± 16.7
NEAP (mEq/d)	86.8 ± 53.9	90.4 ± 58.9	83.8 ± 49.1
DBP (mmHg)	131.0 ± 21.2	132.6 ± 20.1	129.6 ± 22.0
SBP (mmHg)	77.4 ± 11.5	79.1 ± 11.3	75.9 ± 11.5
Sedentary leisure time (h/d)	4.9 ± 2.8	5.1 ± 2.8	4.8 ± 2.7
Physical activity time (h/d)	1.1 ± 0.8	1.1 ± 0.8	1.0 ± 0.7
Hypertension (N, %)	1076(30.7)	550(29.1)	526(32.7)
Smoking (N, %)	2546(72.7)	1842(97.3)	704(43.8)
Alcohol (N, %)	2171(62.0)	1420(75.0)	751(46.7)
Education (N, %)			
*≤ 6 years*	1674(47.8)	1062(56.1)	612(38.1)
*7–12 years*	1482(43.3)	761(40.2)	924(57.5)
*≥ 13 years*	345(9.9)	70(3.7)	72(4.5)
Marital status (N, %)			
*Unmarried*	329(9.4)	178(9.4)	151(9.4)
*Married*	3172(90.6)	1715(90.6)	1457(90.6)

Footprint: *BMI* Body mass index, *PRAL* Potential renal acid load, *NEAP* Net endogenous acid production, *SBP* Systolic blood pressure, *DBP* Diastolic blood pressure

**Fig. 1:**
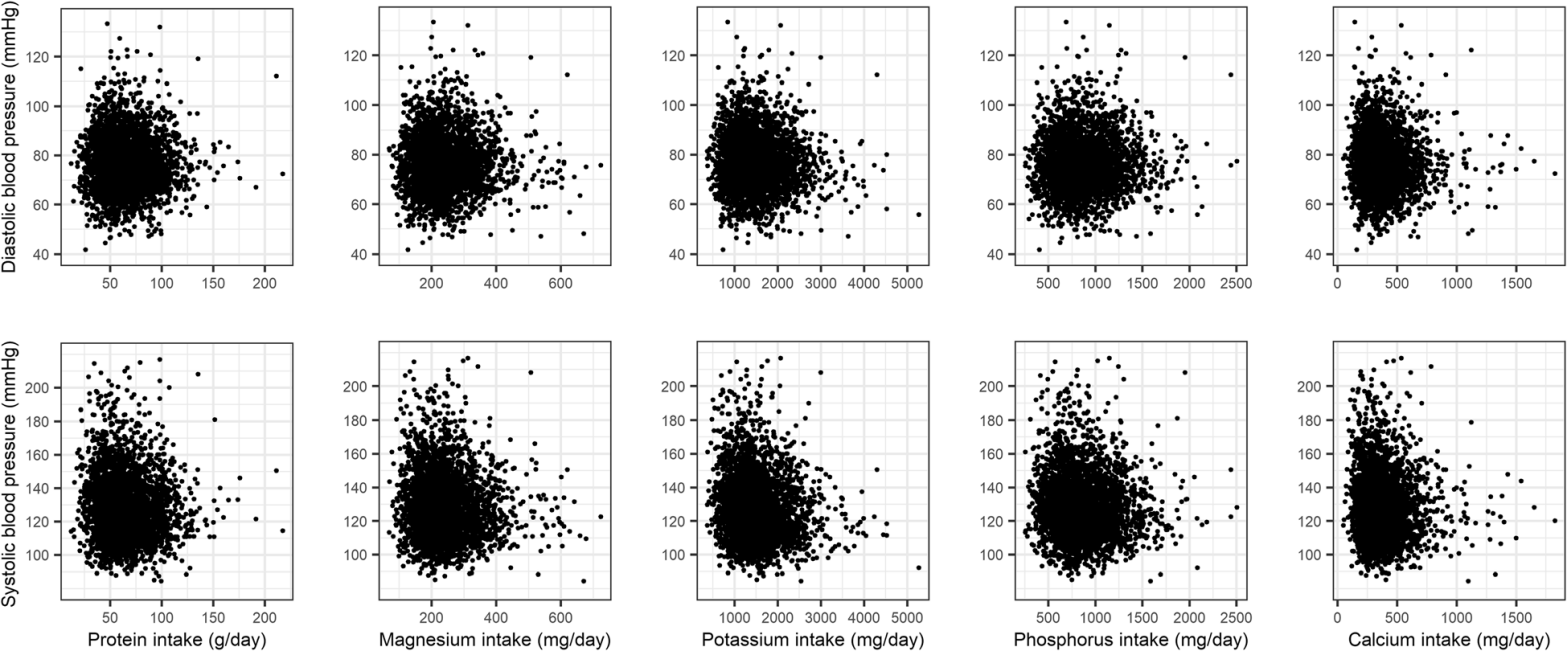
Scatter plots regarding association between blood pressure and five nutrients

Scatter plots did not show obvious linear relationship between blood pressure (SBP and DBP) and different nutrients intake (protein, calcium, potassium, phosphorus and magnesium), as exhibited in Fig. 1.

### Association between PRAL and hypertension

We employed data from the first quartile (Q1) as reference group, crude ORs of the second quartile (Q2), the third quartile (Q3) and the fourth quartile (Q4) were 1.00, 1.10 and 1.05, respectively, which were lack of statistical significance (*P*-values > 0.05 and P-trend = 0.13). After adjusting for sex and age, the associations between PRAL and hypertension were still not statistically significant. In addition, after adjusting for a broad of potential confounding factors including age, sex, smoking, drinking, BMI, sedentary leisure time, physical activity time, sodium intake, education and marital status, the non-significant results remain (Table 2).

**Table 2:** Association between potential renal acid load and the risk of hypertension

Quartiles	n	Crude model			Adjusted model 1^a^			Adjusted model 2^b^		
		OR	95%CI	P	OR	95%CI	P	OR	95%CI	P
Q1	809	Ref.	–	–	Ref.	–	–	Ref.	–	–
Q2	810	1.00	0.80–1.27	0.963	1.02	0.80–1.29	0.894	1.03	0.81–1.31	0.805
Q3	809	1.10	0.87–1.40	0.425	1.05	0.82–1.34	0.680	1.09	0.85–1.40	0.488
Q4	809	1.05	0.83–1.34	0.657	1.11	0.87–1.42	0.407	1.15	0.90–1.49	0.255

Footprint: a = Generalized linear mixed effects model adjusted for sex and age; b = Generalized linear mixed effects model adjusted for sex, age, smoking, drinking, body mass index, sedentary leisure time, physical activity time, sodium intake, education and marital status

**Fig. 2:**
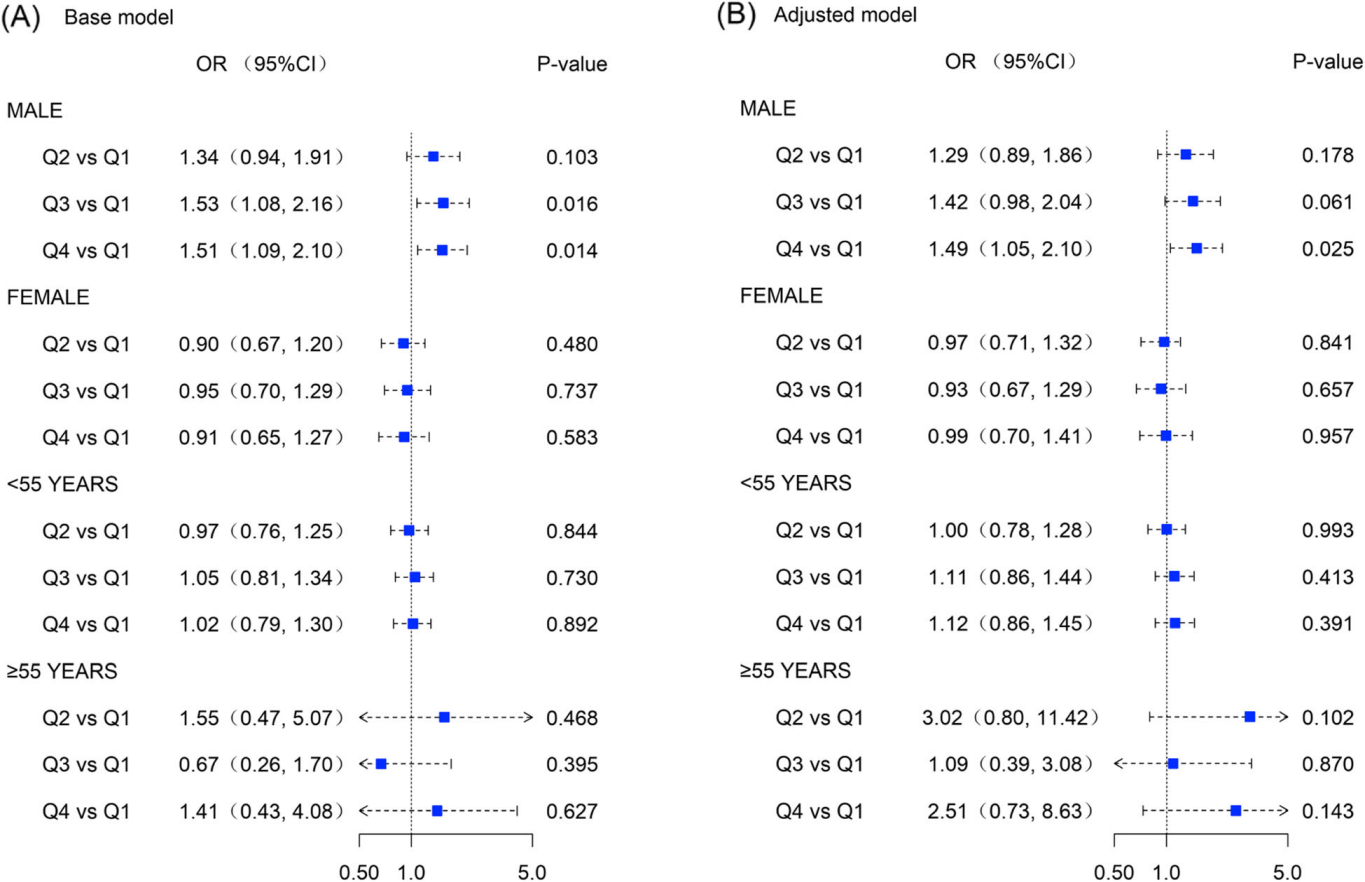
Forest plots regarding association between potential renal acid load and the risk of hypertension. (Plot **a**. assuming base model and Plot **b**. assuming adjusted model)

Results from subgroup analysis indicated that higher PRAL was associated with higher prevalence rate of hypertension among male adults (P-trend = 0.03). Crude OR for Q2 was 1.34 (95%CI, 0.94–1.91), Q3 was 1.53 (95%CI = 1.08, 2.16) and Q4 was 1.51 (95%CI, 1.08–2.16). However, these ORs had a reduction after adjusting for a broad of potential confounding factors (adjusted ORs for Q2, Q3 and Q4 were 1.29, 1.42 and 1.49 respectively). With respect to the female or separated age groups (≤55 years and > 55 years), the associations between elevated PRAL and hypertension risk were not pronounced (Fig. 2).

### Association between NEAP and hypertension

In terms to association between NEAP and hypertension, crude ORs of Q2, Q3 and Q4 were 1.19, 1.16 and 1.24, respectively, which were not statistically significant (*P*-values > 0.05 and P-trend = 0.76). Both adjusted model 1 and adjusted model 2 suggest non-significant results (Table 3).

**Table 3:** Association between potential renal acid load and the risk of hypertension

Quartiles	n	Crude model			Adjusted model 1^a^			Adjusted model 2^b^		
		OR	95%CI	P	OR	95%CI	P	OR	95%CI	P
Q1	808	Ref.	–	–	Ref.	–	–	Ref.	–	–
Q2	809	1.19	0.94–1.51	0.157	1.18	0.92–1.50	0.193	1.12	0.87–1.43	0.388
Q3	807	1.16	0.91–1.47	0.237	1.20	0.94–1.54	0.142	1.12	0.87–1.45	0.367
Q4	809	1.24	0.98–1.57	0.084	1.22	0.95–1.56	0.114	1.10	0.85–1.41	0.484

Footprint: a = Generalized linear mixed effects model adjusted for sex and age; b = Generalized linear mixed effects model adjusted for sex, age, smoking, drinking, body mass index, sedentary leisure time, physical activity time, sodium intake, education and marital status

**Fig. 3:**
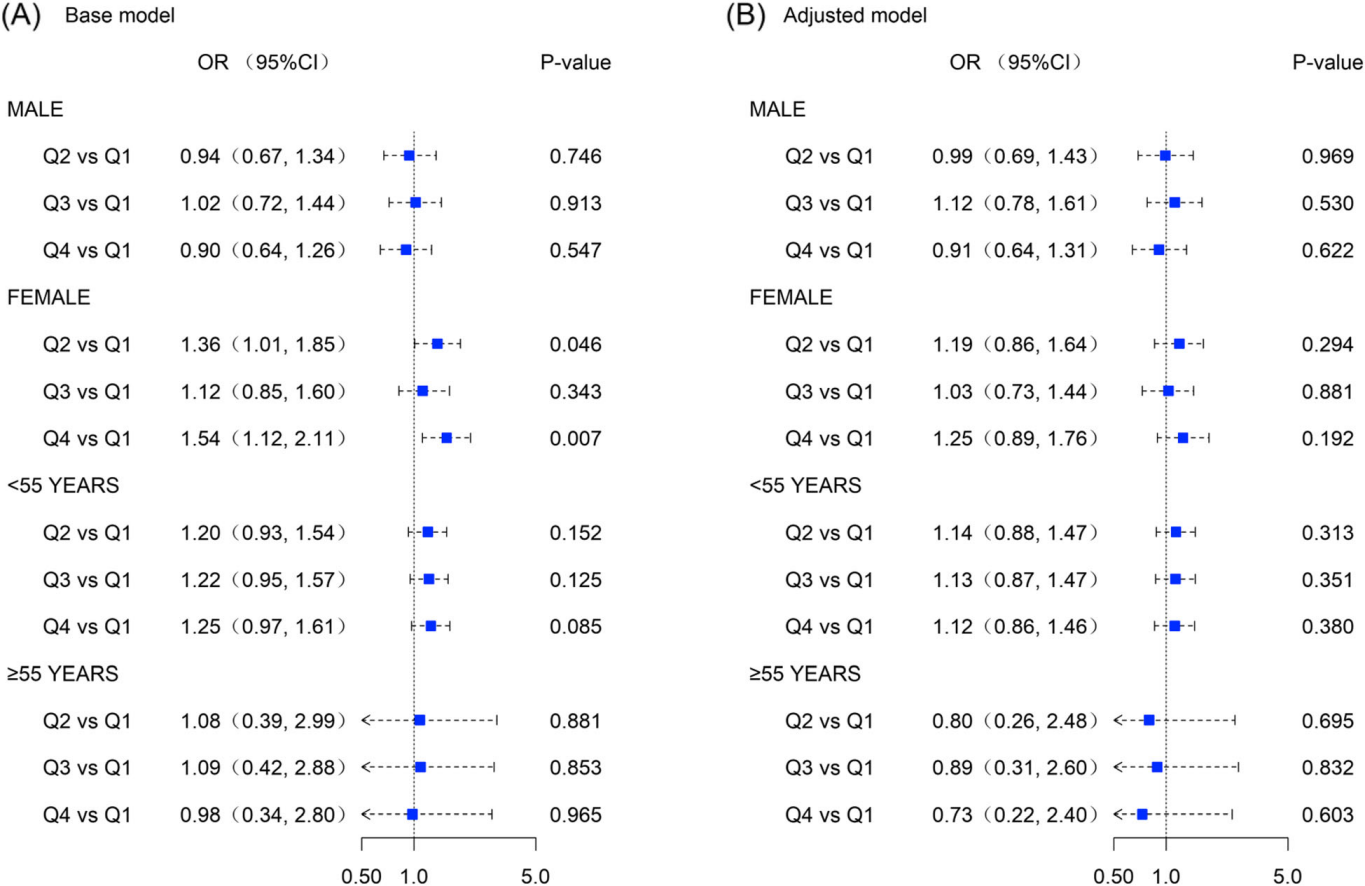
Forest plots regarding association between net endogenous acid production and the risk of hypertension. (Plot **a**. assuming base model and Plot **b**. assuming adjusted model)

Subgroup analysis demonstrated that the associations between elevated NEAP and hypertension risk were not sound among different genders and different age groups (Fig. 3).

## Discussion

To the best of our knowledge, this is the first study from mainland China to evaluate the association between DAL and hypertension in adults using data from large-scale samples of 3501 population. Our analysis showed higher PRAL was associated with higher prevalence rate of hypertension among male adults. However, for the total participants, the female, the participants with ≤55 years or participants with > 55 years, the associations was lack of significance. With respect to association between NEAP and hypertension, non-significant results were identified.

Debates concerning the association between DAL and hypertension were identified in previous studies. Two cohort designed studies from America (comprising 87,293 participants) [27] and Hong Kong, China (comprising 3956 participants) [28] reported an increased risk ratio of hypertension in those with elevated DAL. However, two other cohort studies from Netherlands (comprising 3411 and 2241 participants, respectively) [22, 29] demonstrated that the association was lack of significance. The controversy also exists in cross-sectional studies conducted in Japan [14], Germany [20], Korea [30] and Sweden [31].

Different levels of DAL in various studies might be one of contributors to inconsistent results. Krupp et al. [20] found a 1.45-fold higher prevalence rate of hypertension in participants with higher PRAL (15.5 mEq/day) comparing to a lower PRAL values (− 30.8 mEq/day). Engberink et al. [22] did not identify a significant association between higher PRAL and the risk of hypertension incidence (the medians of lower and higher PRAL were − 14.6 to 19.9 mEq/d, respectively). The medians of lower and higher PRAL in our study (13.81 to 28.19 mEq/d, respectively) also differed from other studies. The other possible explanations might include different study design and inclusion criteria of participants.

Our findings demonstrated higher PRAL was associated with higher prevalence rate of hypertension among male adults, while no significant association was found among female adults. Previous studies have reported some biological factors such as sex hormones, immune inflammatory factors and chromosomal differences [32], which are considered to be protective for the female. Even though the prevalence rate of hypertension in male (29.1%) was mildly lower than female (32.7%), our finding showed a significantly higher PRAL in the male (25.3 ± 20.2 mEq/d) comparing to the female (19.4 ± 16.7 mEq/d). It indicated that blood pressure in the male are more susceptible to PRAL than the female. The discrepancy of DAL in different genders is need to be verified as it disappeared when it comes to the effect of NEAP.

There are uncertain about the mechanism underlying the putative association between elevated DAL and the risk of hypertension, nevertheless, some previous studies were dedicated to explaining the potential relationship. A chronic and low grade metabolic acidosis was supposed to be a main contributor to the risk of hypertension on this issue [22]. The chronic symptoms are characterized with increased proton load and decreased pH value in blood, resulting from a high DAL [20, 31]. Metabolic acidosis could indirectly raise blood pressure through upgrading secreting cortisol, excreting calcium or inhibiting citrate excretion [20, 31]. Another mechanism involving serum anion gap in metabolic acidosis is also discussed. Previous studies demonstrated an elevated blood pressures in individuals with a high level of anion gap [33, 34], nonetheless the possible underlying pathway is not understood. Even though the evidence mentioned above have been confirmed by a set of cross-sectional and animal studies, the possible mechanisms are considered to be weak.

There were several strengths in this study as follows: (1) a large sample size of participants was randomly selected by well-designed sampling strategy; (2) anthropometry was measured by well-trained staff, but not self-reported. However, some potential limitations should also be discussed. First of all, this study was a cross-sectional designed study, which was indeterminately chronological order of the relation and might introduce potential confounding bias. The causal link between dietary acid load and the risk hypertension cannot be confirmed as indeterminately chronological order of the relation. Although we put a broad of confounding factors as we could, such as sex, age, smoking, drinking, body mass index, sedentary leisure time, physical activity time, sodium intake, education and marital status, in the generalized linear mixed effects model, confounding effect of some other potential factors, such as family history, might exist as well. What’s more, information bias might be not eliminate due to a short period of dietary data collection (consecutive 3 days). The collecting data could not comprehensively reflect a long-term dietary habit of local residents and seasonal factors were not taken into consideration. In addition, even though dietary acid load (PRAL and NEAP) were widely employed in previous studies, it was not directly measured, but computed according to dietary intake.

Two aspects should be noted to future studies. The first one is that a comprehensive review about putative association between elevated dietary acid load and the risk of hypertension should be conducted. Quantitative synthesis of available studies could be performed to obtain a comprehensive result on the issue. The second one is that underlying pathway regarding how dietary acid load has impacts on blood pressure should be uncovered if the putative association has been actually proved.

## Conclusions

In summary, association between elevated PRAL and higher prevalence rate of hypertension among male adults was identified, while in terms to total participants, the female, the participants with ≤55 years or participants with > 55 years, the associations was lack of significance. With respect to association between NEAP and hypertension, non-significant results were identified. Given to this study was cross-sectional design, further studies are warranted to elucidate role of DAL in hypertension risk regarding different genders and the underlying pathway how DAL affects blood pressure should be verified and uncovered.

## Data Availability

The datasets used and/or analyzed during the current study available from the corresponding author on reasonable request.

## Abbreviations

BMI: Body mass index
DAL: Dietary acid load
DBP: Diastolic blood pressure
NEAP: Net endogenous acid production
NHS: Nutrition and Health Survey
PRAL: Potential renal acid load
Q1: The first quartile point
Q2: The second quartile point
Q3: The third quartile point
Q4: The fourth quartile point
SBP: Systolic blood pressure
