# Correction to: Association between dietary acid load and the risk of hypertension among adults from South China: result from nutrition and health survey (2015–2017)

**DOI:** 10.1186/s12889-020-08502-1

**Published:** 2020-03-19

**Authors:** Shao-wei Chen, Gui-yuan Ji, Qi Jiang, Ping Wang, Rui Huang, Wen-jun Ma, Zi-hui Chen, Jie-wen Peng

**Affiliations:** Department of Health Risk Assessment Research Center, Guangdong Provincial Institute of Public Health, Guangdong Provincial Center for Disease Control and Prevention, No. 160 Qunxian Road, Panyu District, Guangzhou, 511430 China

**Correction to: BMC Public Health (2019) 19:1599**

**https://doi.org/10.1186/s12889-019-7985-5**

It was highlighted that in the original article [1] the data on categorical variables was wrongly arranged in Table 1 and there was an incorrect statement in the fourth paragraph of the Discussion section. This Correction article shows the incorrect and correct statement of paragraph 4 and the correct Table 1.

**Table 1:** Characteristic of eligible participants

	Overall (*n* = 3501)	Male (*n* = 1608)	Female (*n* = 1893)
Age (years)	52.0 ± 15.0	52.9 ± 15.2	51.2 ± 14.7
Ethnicity (N, %)			
*Han*	3473 (99.2)	1599 (99.4)	1874 (99.0)
*Other*	28 (0.8)	9 (0.6)	19 (1.0)
BMI (Kg/m2)	23.4 ± 3.5	23.3 ± 3.4	23.4 ± 3.6
Nutrient intake			
*Protein (g/d)*	65.9 ± 36.5	71.5 ± 40.4	61.1 ± 32.0
*Calcium (mg/d)*	1501.6 ± 603.9	1564.6 ± 579.3	1448.1 ± 619.1
*Potassium (mg/d)*	381.1 ± 211.1	389.4 ± 184.7	374.1 ± 231.0
*Phosphorus (mg/d)*	866.6 ± 298.7	926.2 ± 298.9	815.9 ± 289.1
*Magnesium (mg/d)*	238.4 ± 82.3	250.7 ± 83.3	228.0 ± 80.0
*Sodium (mg/d)*	4893.5 ± 3366.0	5228.2 ± 3400.4	4609.2 ± 3310.9
Total energy intake (Kcal/d)	1757.4 ± 566.5	1908.6 ± 604.8	1629.0 ± 497.1
PRAL (mEq/d)	22.1 ± 18.6	25.3 ± 20.2	19.4 ± 16.7
NEAP (mEq/d)	86.8 ± 53.9	90.4 ± 58.9	83.8 ± 49.1
DBP (mmHg)	131.0 ± 21.2	132.6 ± 20.1	129.6 ± 22.0
SBP (mmHg)	77.4 ± 11.5	79.1 ± 11.3	75.9 ± 11.5
Sedentary leisure time (h/d)	4.9 ± 2.8	5.1 ± 2.8	4.8 ± 2.7
Physical activity time (h/d)	1.1 ± 0.8	1.1 ± 0.8	1.0 ± 0.7
Hypertension (N, %)	1076 (30.7)	526 (32.7)	550 (29.1)
Smoking (N, %)	955 (27.3)	904 (56.2)	51 (2.7)
Alcohol (N, %)	1330 (38.0)	857 (53.3)	473 (25.0)
Education (N, %)			
*≤ 6 years*	1674 (47.8)	612 (38.1)	1062 (56.1)
*7–12 years*	1482 (43.3)	924 (57.5)	761 (40.2)
*≥ 13 years*	345 (9.9)	72 (4.5)	70 (3.7)
Marital status (N, %)			
*Unmarried*	329 (9.4)	151 (9.4)	178 (9.4)
*Married*	3172 (90.6)	1457 (90.6)	1715 (90.6)

Footprint: *BMI* body mass index, *PRAL* potential renal acid load, *NEAP* net endogenous acid production, *SBP* systolic blood pressure, *DBP* diastolic blood pressure

**Incorrect statement**

Even though the prevalence rate of hypertension in male (29.1%) was mildly lower than female (32.7%), our finding showed a significantly higher PRAL in the male (25.3 ± 20.2 mEq/d) comparing to the female (19.4 ± 16.7 mEq/d).

**Correct statement**

Prevalence rate of hypertension in male (32.7%) was mildly higher than female (29.1%) and a significantly higher PRAL in the male (25.3 ± 20.2 mEq/d) was also identified comparing to the female (19.4 ± 16.7 mEq/d).
